# Baicalein Neutralizes Hypercholesterolemia-Induced Aggravation of Oxidative Injury in Rats

**DOI:** 10.7150/ijms.46108

**Published:** 2020-05-18

**Authors:** Abdulaziz MS AlSaad, Mohamed Mohany, Mohammed S Almalki, Ibrahim Almutham, Abdulwahab A Alahmari, Mohammed AlSulaiman, Salim S Al-Rejaie

**Affiliations:** Department of Pharmacology and Toxicology, College of Pharmacy, King Saud University, P.O. Box 55760, Riyadh - 1145, Saudi Arabia

**Keywords:** hypercholesterolemia, baicalein, inflammation, oxidative stress

## Abstract

Hypercholesterolemia is a major risk factor for several cardiovascular and metabolic diseases as it triggers oxidative and pro-inflammatory cascades. Baicalein (BL) is a natural flavone with multiple therapeutic properties. The present study aimed to evaluate the potential protective effect of BL supplementation in hypercholesterolaemic rats. Rats were fed a high-cholesterol diet (HCD) for six weeks and then orally administered BL at two doses (25 and 50 mg/kg body weight/day) for four weeks. Serum lipids, liver enzymes, cardiac enzymes, renal markers, tumor necrosis factor-α (TNF-α), interleukin-6 (IL-6), interleukin-1β (IL-1β), interleukin-10 (IL-10), caspase-3, nitric oxide (NO) and prostaglandin-2 (PGE-2) were measured. In renal, hepatic, and cardiac tissues, thiobarbituric acid-reactive (TBARS) substance, glutathione (GSH), superoxide dismutase (SOD), catalase (CAT), and glutathione peroxidase (GPx) activities were measured. The altered levels of lipoproteins, aminotransferases, creatine kinases, and urea in hypercholesterolemic animals were significantly corrected by BL. Inflammatory and apoptotic biomarkers were also markedly attenuated in the HCD group following BL treatment. Hypercholesterolemia considerably induced the lipid peroxidation product, TBARS, and oxidative radicals in cardiac, hepatic, and renal tissues, which were attenuated by BL treatment, particularly, at the 50 mg/kg/day dose. BL enhanced the activities of superoxide dismutase, catalase, and glutathione peroxidase that were suppressed by HCD. Histological alterations induced by cholesterol overload in cardiac, hepatic, and renal tissues were ameliorated by BL supplementation. Our results show that the BL treatments (25 and 50 mg/kg/day) to HCD fed rats improved all the altered parameters. These results demonstrate that BL treatment improves cardiac, renal and hepatic dysfunctions in hypercholesterolaemic rats by activation of cellular antioxidant enzymes and/or suppression of inflammatory cytokines.

## Introduction

Hypercholesterolemia is a major global health problem. Epidemiological studies showed that the incidence of hypercholesterolemia is mainly associated with poor dietary habits, such as the consumption of foods containing excessive saturated fats and cholesterol, as well as a lack of exercise. The incidence of hypercholesterolemia is higher in women than in men 1. The World Health Organization reported approximately 2.6 million deaths due to hypercholesterolemia 2. Hypercholesterolemia has multiple significant consequences on different physiological systems, and is one of the major risk factors for several health problems, including ischemic heart diseases, fatty liver, and kidney diseases 3-5. Altered cardiac systolic and diastolic functions as well as contractile dysfunction have been reported in rodents that were fed a high-cholesterol diet (HCD) 6. Basal cardiac autophagy was recently demonstrated to be suppressed by hypercholesterolemia in rats 7. Hypercholesterolemia reportedly triggers lipid accumulation in the liver that negatively influences hepatic functions 8, 9. Increased cholesterol intake impairs renal functions and provokes kidney damage in rodents 10.

Several molecular pathways have been investigated to explore the mechanisms underlying hypercholesterolemia-associated metabolic disturbances. Among the contributing mechanisms, overproduction of reactive oxygen species (ROS) and consequent oxidative stress are commonly documented 11. Numerous experimental studies have reported that cholesterol overload markedly induces ROS accumulation and redox imbalance in tissues. Lipid peroxidation of cellular membranes has also been implicated as a causative mechanism 12. Moreover, studies have revealed links between oxidative stress and inflammation that were closely correlated with tissue necrosis and cellular apoptosis during hypercholesterolemia. Biomarkers of inflammation and programmed DNA damage were found to be elevated by HCD in rodents 11. Activation of nuclear factor-kappa B (NF-κB) and similar transcription factors as well as generation of oxidized low-density lipoprotein may explain this correlation 13.

The potential therapeutic effects of phytochemicals, such as flavonoids, in metabolic disorders associated with hypercholesterolemia have been evaluated in various studies 11, 14. Baicalein (BL) is a 5,6,7-trihydroxyflavone isolated from *Scutellaria* species. BL is known for its multiple pharmacological properties, such as antioxidant and anti-inflammatory effects, in several disorders like cancer and cardiac, neurological, hepatic, and renal diseases 15, 16. In addition, studies have shown the ability of BL to ameliorate diabetes-associated metabolic complications via suppression of hyperglycemia, inflammation, free-radical production, and NF-κB-related pathways 17. A recent study revealed that BL might provide effective protection against oxidized low-density lipoprotein-induced oxidative and inflammatory damage 18. Therefore, the present study aimed to explore the potential protective role of BL on metabolism and redox status in rats fed a HCD.

## Materials and Methods

### Animals

Male albino Wistar rats (70-80 g) were obtained from the Pharmacy College Animal Care Center at King Saud University. The animals were acclimatized for 10 days (during this period animal weights become 100-110 g) prior to starting the experiments. In this study, young animals were selected as cholesterol diet effects are more prominent in young ages than old 19. In our earlier HCD-fed rats models we used similar age and weight of Wistar rats 20, 21 The rats were housed in standard conditions of 22 ± 1°C, 50-55% humidity, and 12-h day/night cycles. All experimental protocols, including euthanasia procedure, blood sampling, and final sacrifice followed National Institutes of Health guidelines on the care and use of laboratory animals (NIH, 1996), and this animal study was approved by the Ethical Committee of Pharmacy College, Animal Care Center, King Saud University.

### Diets

HCD in pellet form was prepared weekly by adding 1% cholesterol + 0.5% cholic acid to normal cholesterol rat chow powder (protein 20%, fat 4%, fiber 3.5%, ash 6%, total energy 2850 Kcal/kg) in our laboratory, shade dried and stored in cool and dry place. Six rats were fed normal cholesterol rat chow, and eighteen rats were fed HCF for 6 weeks. The rats had free access to water and food throughout the experimental period.

### Experimental design

After six weeks, the HCD-fed rats were randomly divided into three groups (n = 6 rats in each group). The four treatment groups in this study were as follows: Group-1, rats fed normal rat chow and treated with vehicle (control group); Group-2, HCD-fed rats treated with vehicle; Group-3, HCD-fed rats treated with BL (25 mg/kg/day, orally, “low dose”) for four weeks; Group-4, HCD-fed rats treated with BL (50 mg/kg/day, orally, “high dose”) for four weeks. HCD feeding was continued during BL supplementation until the end of experiment. Body weight and general health conditions were carefully monitored weekly throughout the experimental period. Blood samples were collected by cardiac puncture under light ether anesthesia and were centrifuged at 4,000 rpm for 10 min; the serum samples were stored at -20°C until analysis. At the end of the experimental period, animals were decapitated and heart, liver, and kidneys were dissected, and weighed. A small portion of the tissues was immediately dipped into liquid nitrogen for 1 min and then stored at -80°C until analysis. Heart, liver, and kidney tissues were preserved in 10% formaldehyde for histopathological evaluations.

### Serum analyses

Total cholesterol (TC), triglycerides (TG), low-density lipoprotein-cholesterol (LDL), high-density lipoprotein-cholesterol (HDL), creatinine, and blood urea nitrogen (BUN) levels were estimated using commercially available diagnostic kits (Human Diagnostics, Wiesbaden, Germany).The serum activities of creatine kinase-B (CK-B), lactate dehydrogenase (LDH), creatine kinase-MB (CK-MB), alanine aminotransferase (ALT), aspartate aminotransferase (AST) were measured using commercially available diagnostic kits (Human Diagnostics, Wiesbaden, Germany). Inflammatory biomarkers, including TNF-α, IL-1β, IL-6, IL-10, PGE-2, caspase-3, NO and NF-κB were measured using ELISA kits for rats (R&D Systems, USA).

### Tissue analyses

Organ's (heart, liver and kidney) small portions were homogenized in physiological buffer (1:10, w/v) and TBARS and GSH levels were measured by using ELISA kits (Cayman Chemical Co., USA). In Post-mitochondria supernatants of heart, liver and kidney, enzymatic activities of SOD, CAT and GPx were measured by using ELISA kits (R&D systems Inc., USA).

### Histopathological procedures

Across sectional portion of a heart, liver and kidney tissues from each group of treatment were preserved in 10% buffered formalin. The samples were embedded in paraffin blocks and sections of thickness 5 μm were cut using a Leica CM3050 S Research Cryostat (Leica Bio-systems, USA). The sections were stained with H&E. Finally, they were examined under the microscope for histopathological changes by an observer who was blind with respect to the treatment groups.

### Statistical analysis

Data are expressed as the mean ± standard error of the mean (SEM) and were analyzed using one-way analysis of variance (ANOVA) followed by Student-Newman-Keuls multiple comparison tests (*n* = 6). Differences between groups were considered statistically significant when *P* ≤ 0.05. All statistical analyses were conducted using Graph-Pad Prism (v. 5) software.

## Results

Serum lipid profile is presented in table 1. In HCD fed rats, TC, TG and LDL levels were significantly (P<0.001) increased compared to control animals. BL treatment to hypercholesteremic rats markedly reduced the TG and TC levels were significantly P<0.05 and P<0.01 inhibited in BL (25 and 50 mg/kg/day) treated groups as compared to HCD group of rats respectively. The high dose of BL (50 mg/kg/day) only inhibited the TC levels significantly (P<0.05) compared to HCD group. However, HDL levels did not markedly alter in HCD group when compared to controls (Table 1). The enzymes of CK, CK-MB and LDH are considered the cardiac markers and these were estimated and shown in Table 1. In HCF administered rats, the serum enzymes of CK, CK-MB and LDH were shown to increases (P<0.001) compared to control group. BL (50 mg/kg/day) treatment showed significant (P<0.05) inhibition in enzymatic activity of LDH compared to HCD. The CK and CK-MB levels were markedly reduced by both the doses of BL (Table 1).

**Figure 1:**
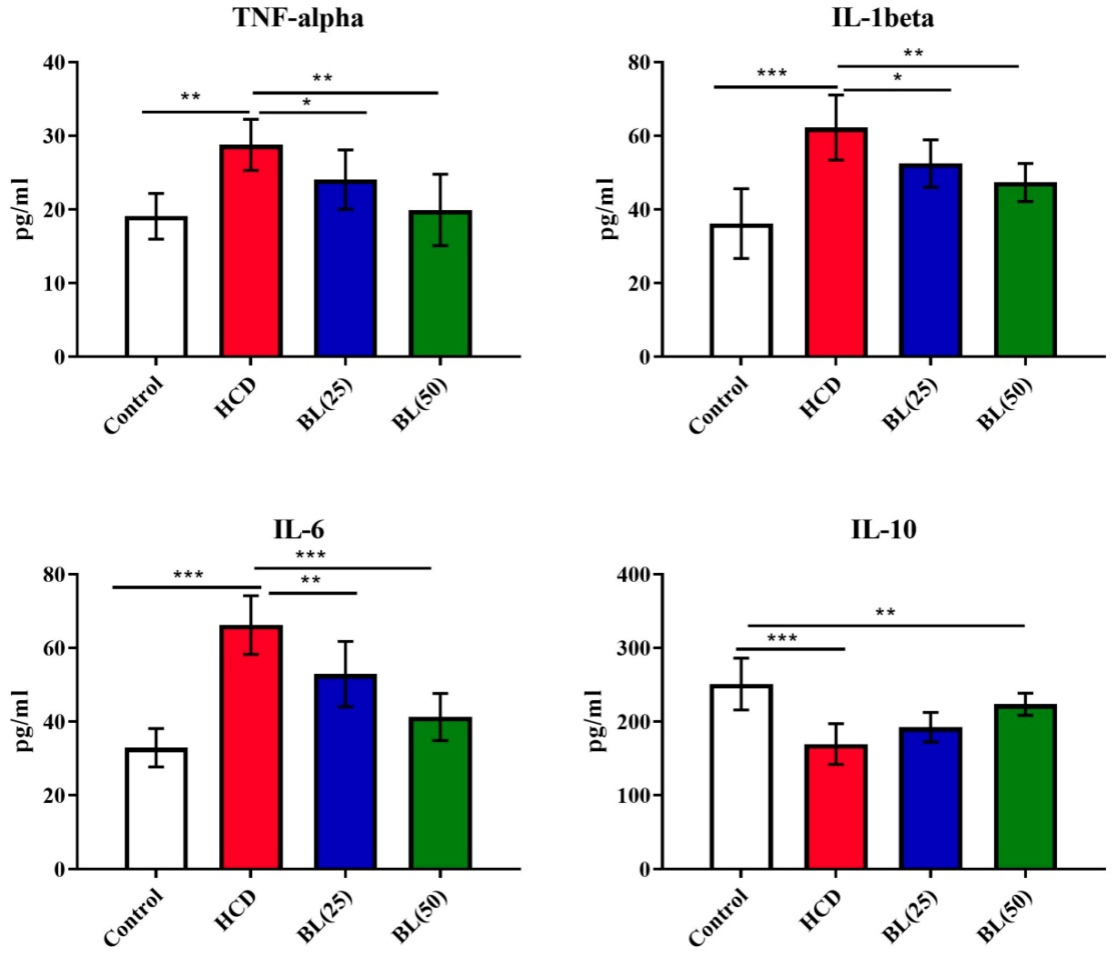
Effect of BL on hypercholesterolemia-induced changes in serum inflammatory biomarkers including tumor necrosis factor-alpha (TNF-α), interleukin-6 (IL-6), interleukin-1beta (IL-1β) and interleukin-10 (IL-10). Data are expressed as the mean ± SEM (*n*= 6 per group). Statistically significant difference was considered at ^*^p < 0.05, ^**^p < 0.01and ^***^p < 0.001.

**Figure 2:**
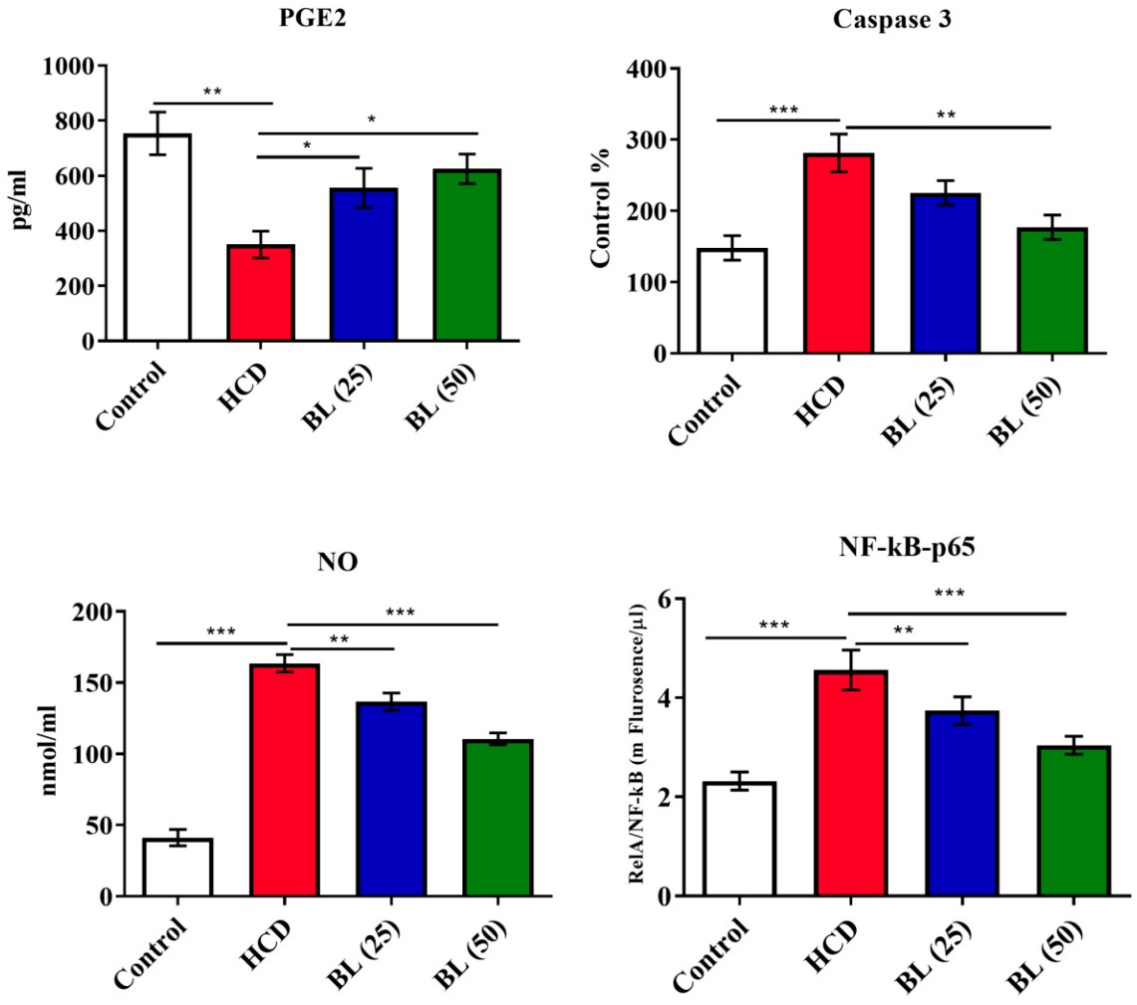
Effect of BL on hypercholesterolemia-induced changes in serum prostaglandin E-2 (PGE-2), Caspase 3, nitric oxide (NO) and NF-κB levels. Data are expressed as the mean ± SEM (*n*= 6 per group). Statistically significant difference was considered at ^*^p < 0.05, ^**^p < 0.01and ^***^p < 0.001.

Serum levels of TNF-α, IL-6 and IL-1β were significantly (P<0.001) elevated, while IL-10 levels reduced (P<0.001) in HCD fed animals compared to control rats. BL treatment to hypercholesteremic rats for four weeks markedly reduced the pro-inflammatory cytokines in dose dependent manner. The anti-inflammatory cytokine IL-10 markedly (P<0.01) elevated in BL (50 mg/kg/day) treated group **(**Figure 1**)**. Similarly, the levels of caspase 3, NO as well as NF-κB activity were significantly (P<0.001) increased, while PGE-2 levels were significantly (P<0.001) reduced in HCD group. BL treatment markedly corrected (P<0.01) the altered levels and activity of caspase 3, NO, NF-κB and PGE-2 as compared with HCD group (Figure 2).

**Figure 3:**
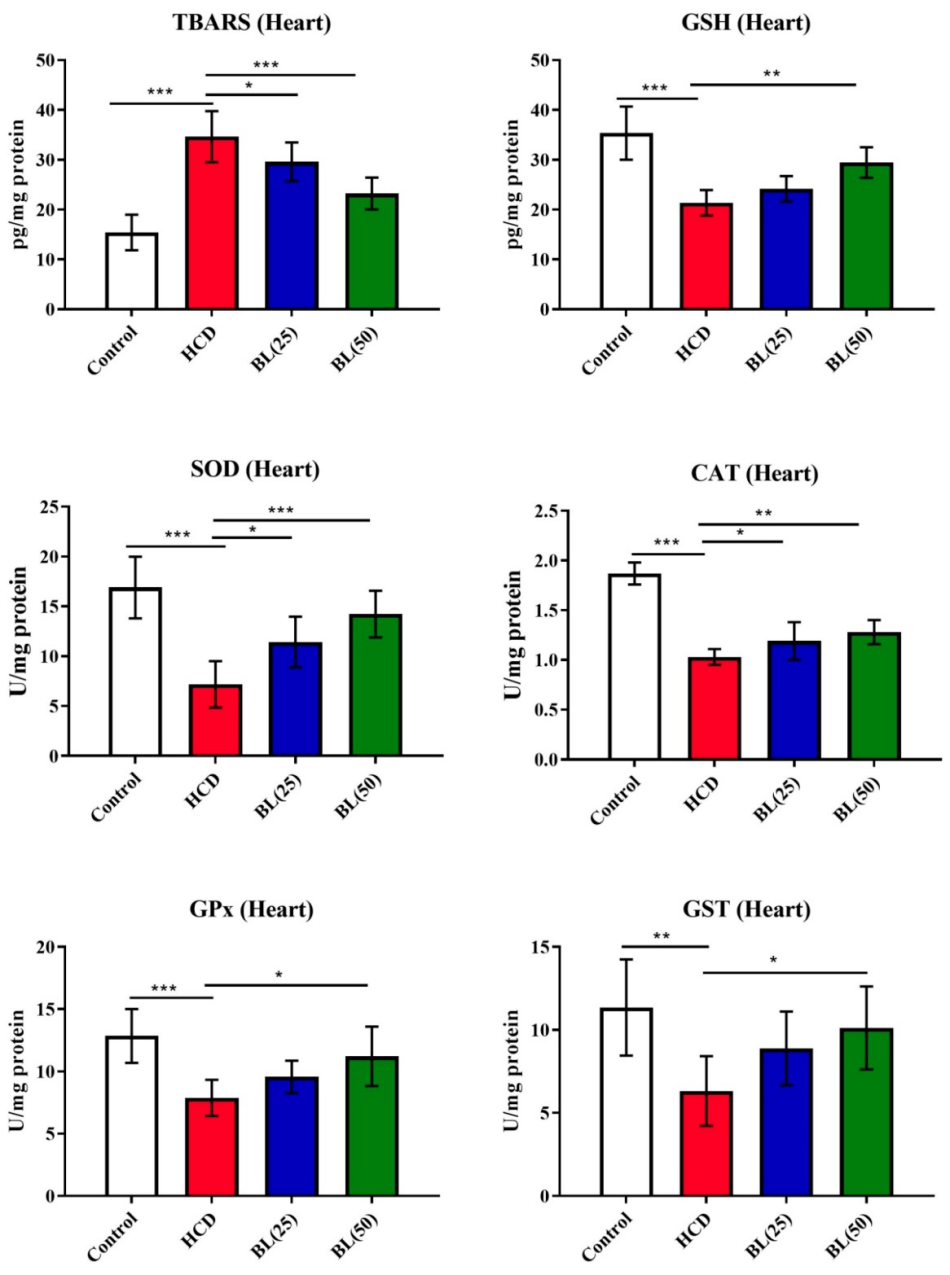
Effect of BL on hypercholesterolemia-induced thiobarbituric reactive substances (TBARS) and glutathione (GSH) levels, and enzymatic activities of superoxide dismutase (SOD), catalase (CAT), glutathione-S-transferase (GST) and glutathione oxidase (GPx) in cardiac tissue. Data are expressed as the mean ± SEM (*n*= 6 per group). Statistically significant difference was considered at ^*^p < 0.05, ^**^p < 0.01and ^***^p < 0.001.

TBARS level was in high significantly (P<0.001) while GSH level was reduced (P<0.001) in heart tissue of HCD fed rats compared to control animals. BL treatment (25 and 50 mg/kg/day) for 4 weeks to HCF fed rats, the TBARS was reduced markedly (P<0.05 and P<0.001, respectively) and the GSH was increased (P<0.01) in BL (50 mg/kg/day) treated group when compared to HCF supplemented control rats. Enzymatic cardiac antioxidants of SOD, CAT and GPx were found to reduces (P<0.001) in HCF fed rats compared to control group. Both the doses of BL markedly (P<0.05 and P<0.01, respectively) enhanced the enzymatic activities of SOD and CAT compared to HCD group. While the enzymatic activity of GPx was markedly elevated in BL (50 mg/kg/day) treated group (Figure 3).

**Figure 4:**
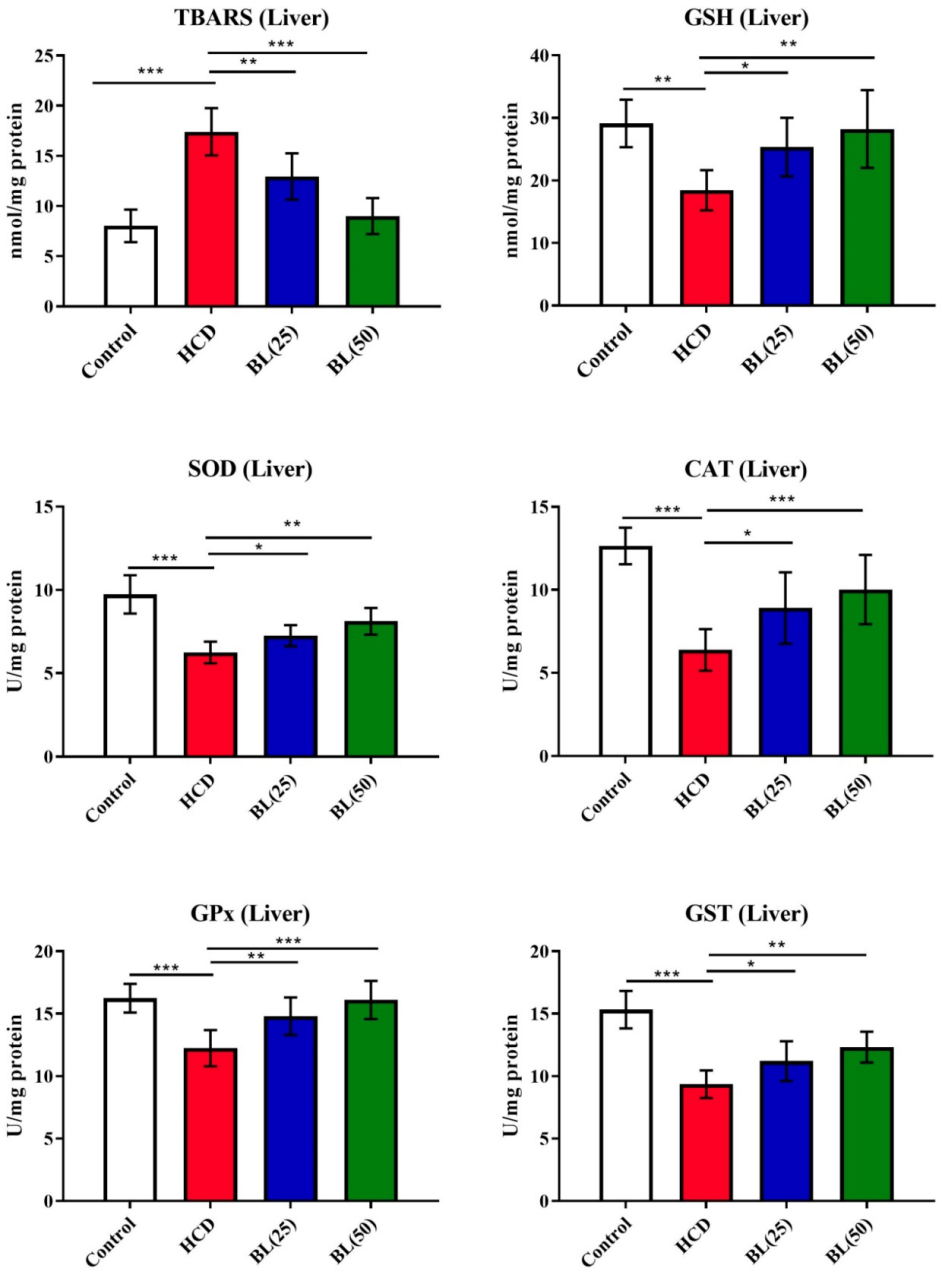
Effect of BL on hypercholesterolemia-induced thiobarbituric reactive substances (TBARS) and glutathione (GSH) levels, and enzymatic activities of superoxide dismutase (SOD), catalase (CAT), glutathione-S-transferase (GST) and glutathione oxidase (GPx) in hepatic tissue. Data are expressed as the mean ± SEM (*n*= 6 per group). Statistically significant difference was considered at ^*^p < 0.05, ^**^p < 0.01and ^***^p < 0.001.

TBARS levels were significantly (P<0.001) increased in hepatic tissue of HCD fed rats while GSH levels found inhibited markedly (P<0.001) by the HCD supplementation compared to normal healthy control rats. Treatment with BL (25 and 50 mg/kg/day) produced inhibition in TBARS levels (P<0.05 and P<0.0, respectively) compared to HCD group of rats. While, GSH levels were significantly (P<0.05) enhanced by the BL (50 mg/kg/day) treatment. Enzymatic activities of SOD, CAT and GPx were significantly (P<0.001) inhibited in hepatic cells of HCD fed rats compared to control animals. Treatment with BL (25 and 50 mg/kg/day) markedly (P<0.05 and P<0.01, respectively) enhanced the SOD and GPx activities in hepatic cells compared to untreated hypercholesteremic rats. However, the CAT activity was significantly (P<0.05) increased by the BL (50 mg/kg/day) treatment compared to HCD fed rats (Figure 4).

**Figure 5:**
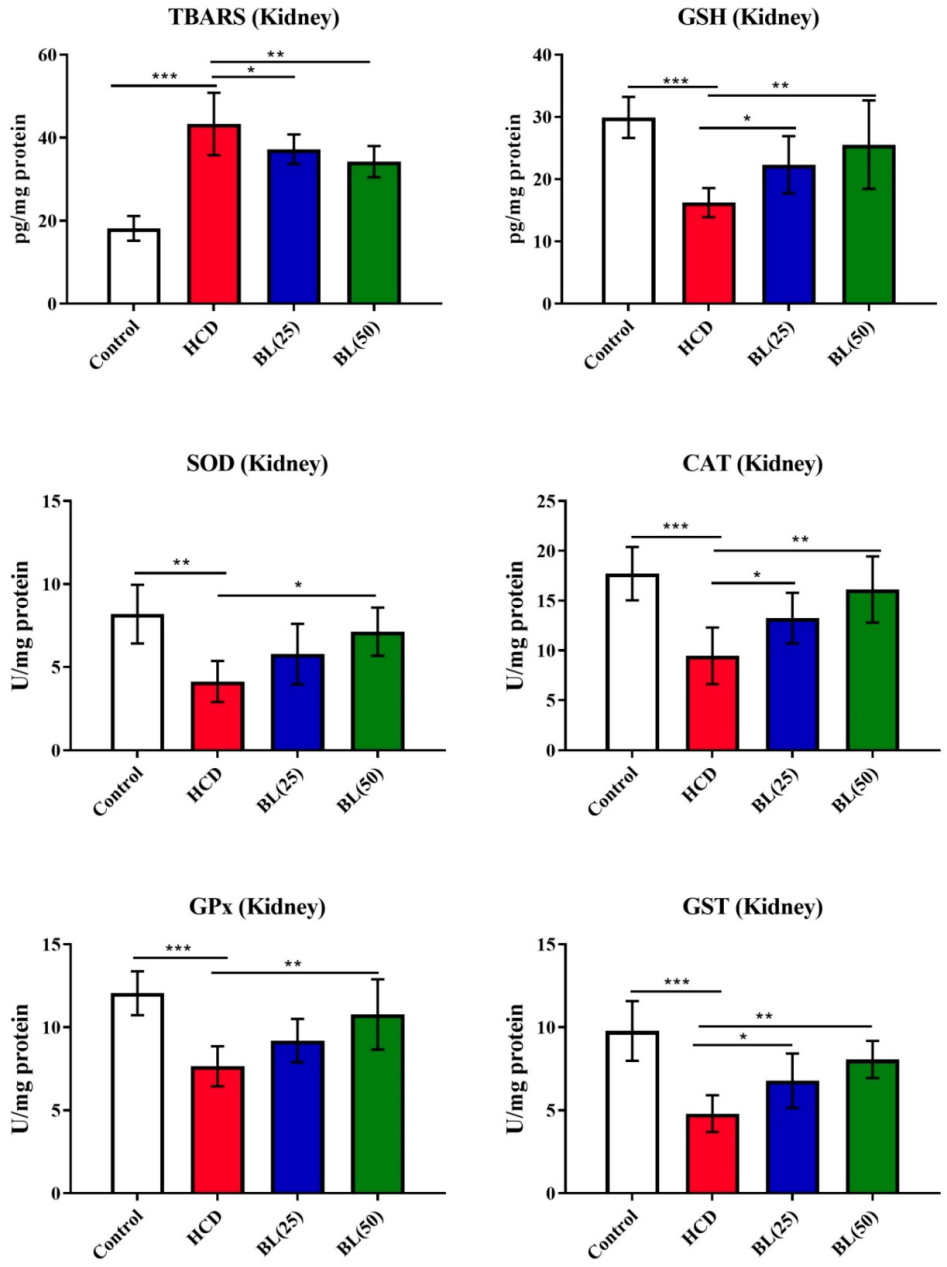
Effect of BL on hypercholesterolemia-induced thiobarbituric reactive substances (TBARS) and glutathione (GSH) levels, and enzymatic activities of superoxide dismutase (SOD), catalase (CAT), glutathione-S-transferase (GST) and glutathione oxidase (GPx) in renal tissue. Data are expressed as the mean ± SEM (*n*= 6 per group). Statistically significant difference was considered at ^*^p < 0.05, ^**^p < 0.01and ^***^p < 0.001.

In kidney, TBARS levels were significantly (P<0.001) increased in hypercholesteremic rats while GSH levels reduced markedly (P<0.001) by the HCD supplementation when compared to normal healthy control rats. Treatment with BL (50 mg/kg/day) produced inhibition (P<0.01) in kidney TBARS levels compared to HCD group. The kidney GSH levels markedly (P<0.01) inhibited by BL treatment (50 mg/kg/day) to HCD fed rats compared to HCD fed untreated animals. Enzymatic activities of SOD (P<0.01), CAT (P<0.01) and GPx (P<0.001) were significantly inhibited in renal tissue of HCD fed rats compared to control animals. BL (50 mg/kg/day) treatment, significantly (P<0.05) enhanced the enzymatic activities of SOD and CAT while GPx activity increased more significantly (P<0.01) in renal tissue compared to untreated hypercholesteremic rats (Figure 5).

**Figure 6:**
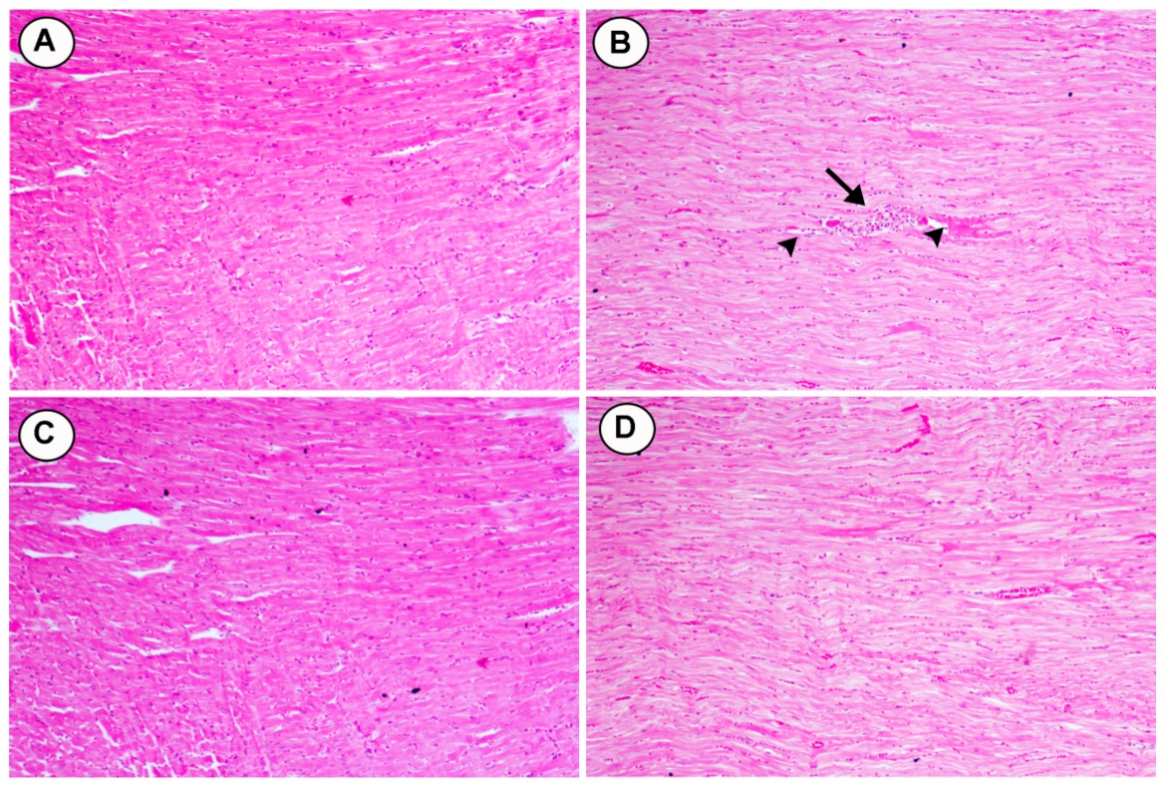
Effects of BL (25 and 50 mg/kg) supplementation on hypercholesterolemia-induced histopathological changes in cardiac tissues (H&E, X100). (A) Section from control group, (B) Section from HCD group with multi-focal vacuolar degeneration (heads- arrow) and mononuclear cell infiltration (arrow), (C and D) Section from BL(25 and 50 mg/kg) group showing improvements in myocardial cell morphology.

Histological changes were seen in cross sections of heart tissues from rats fed HCD and treated with two doses of BL (25 and 50 mg/kg): A) The control group showing the normal appearance of myocardial cells with oval elongated nuclei and homogenous cytoplasm. B) Section of heart tissue from rats feeding HCD showed multi focal vacuolar degeneration (heads- arrow) and congestion of blood capillaries (arrow). C) Moderate myocardial cell morphology with oval-elongate nucleus centrally and homogeneous cytoplasm were shown in myocardiocytes of HCD rats treated with (25 mg/kg). D) Normal myocardial cell morphology with oval-elongate nucleus centrally and homogeneous cytoplasm were shown in myocardiocytes of HCD rats treated with (50 mg/kg) (Figure 6).

**Figure 7:**
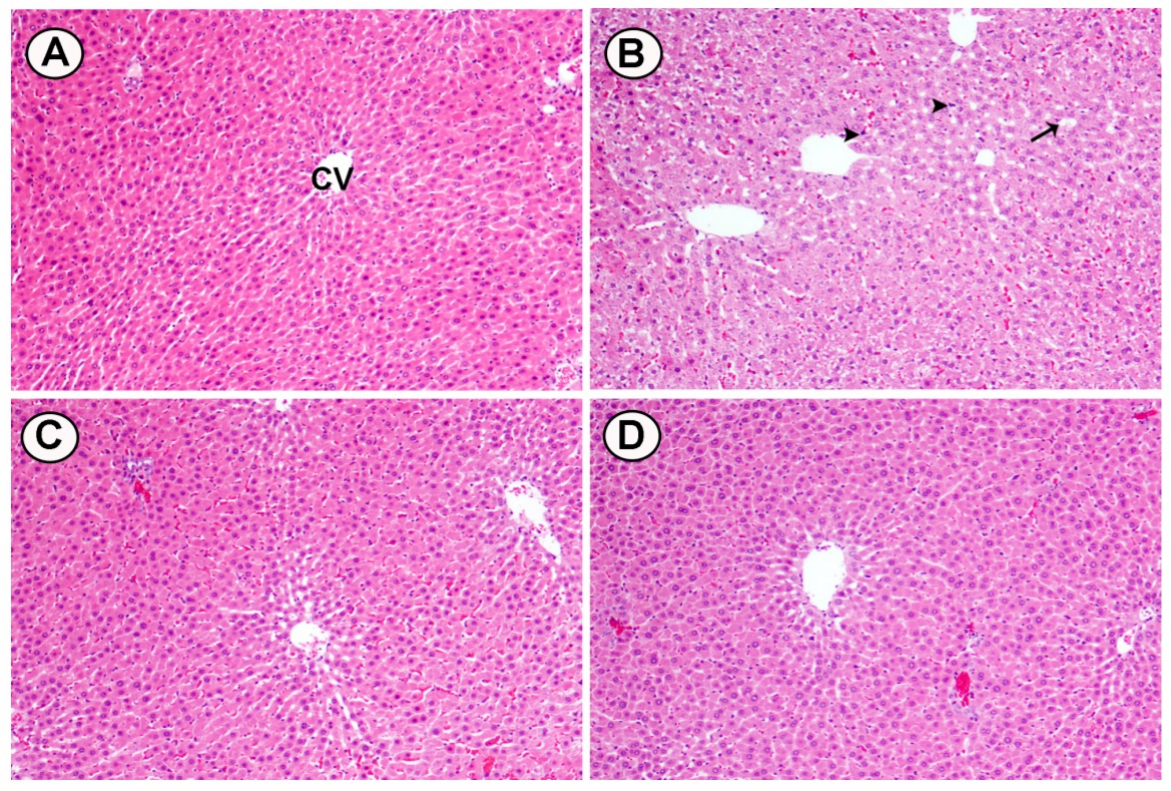
Effects of BL (25 and 50 mg/kg) supplementation on hypercholesterolemia-induced histopathological changes in hepatic tissues. (A) Section from control group, (B) Section from HCD group with marked fat deposition (arrow), dilated sinusoids and pyknotic nuclei (head arrows), (C) Section from BL(25) group showing injury in hepatocytes and less fat deposition and (D) Section from BL(50) group showing moderate injury in hepatocytes and less fat deposition. (H&E, 100X).

Histological changes were seen in cross sections of liver tissues from rats fed HCD and treated with two doses of BL (25 and 50 mg/kg): A) The liver from a control rat shows normal hepatocytes and CV. B) Liver of rats fed high cholesterol showed marked fat deposition (arrow), dilated sinusoids and pyknotic nuclei (head arrow). C) Liver of HCD treated with (25 mg/kg) BL showed moderate injury in hepatocytes and less fat deposition. D) Liver of HCD treated with (50 mg/ kg) BL showed moderate injury in hepatocytes and less fat deposition (Figure 7).

**Figure 8:**
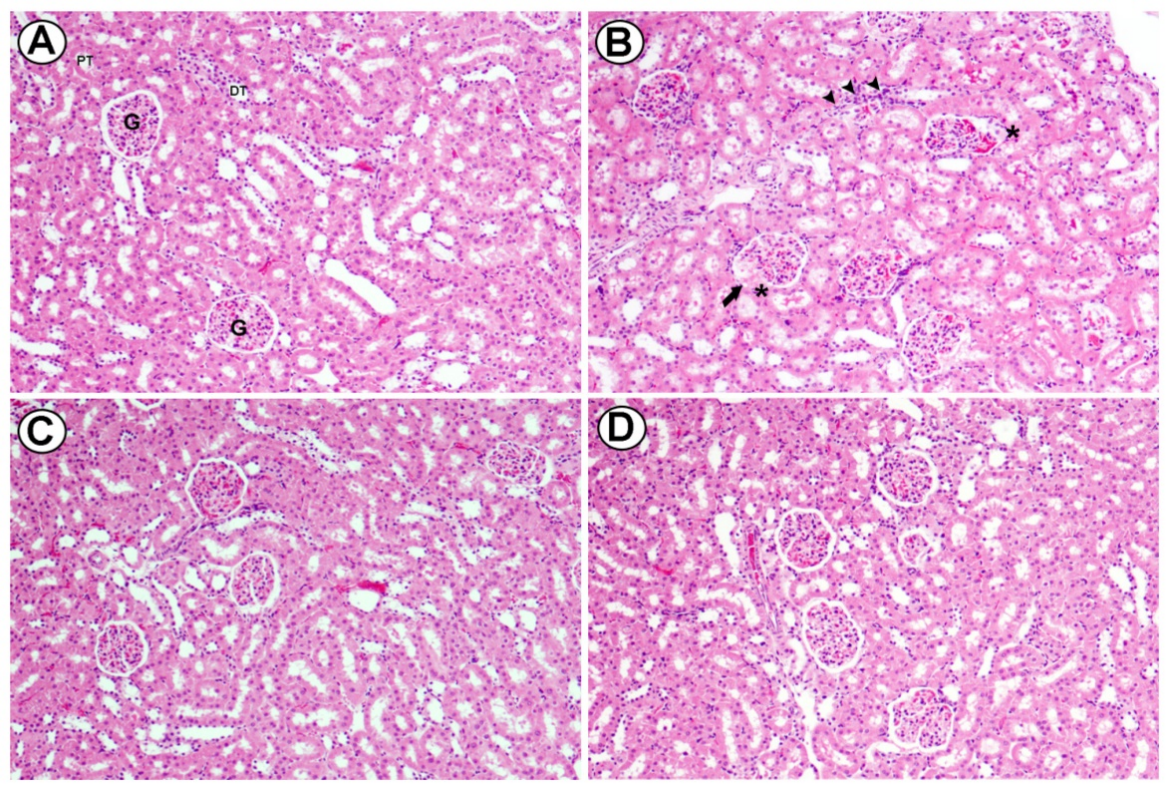
Light micrographs of renal cortex of rats fed high cholesterol diet and administered orally with two doses of Baicalein (25 and 50 mg / Kg bwt.). Section from the renal cortex of the control group reveals the normal appearance of the proximal convoluted tubules (PT), distal convoluted tubules (DT), Bowman's capsule and glomerulus (G) (A). Renal cortex of rats fed high cholesterol showed dilatation in glomerular capillaries (arrow), thickening in basal membrane of glomerulus (asterisks) and interstitial mononuclear cell infiltration was seen (head arrows) (B). Renal cortex of high cholesterol diet treated with (25 mg /Kg bwt, C) and (50 mg / Kg bwt., D) of Baicalein showed reduced injury in glomeruli and renal tubules. H&E, scale bar = 50 µm.

Light micrographs of renal cortex of rats fed high cholesterol diet and administered orally with two doses of Baicalein (25 and 50 mg/kg). Section from the renal cortex of the control group reveals the normal appearance of the PT, DT, Bowman's capsule and glomerulus (G) (A). Renal cortex of rats fed high cholesterol showed dilatation in glomerular capillaries (head arrow), thickening in basal membrane of glomerulus (arrow) and mononuclear cell infiltration was seen (curved arrow) (B). Renal cortex of high cholesterol diet treated with (25 mg/kg) and (50 mg/kg) of Baicalein showed reduced injury in glomeruli and renal tubules. H&E, scale bar = 50 µm. (Figure 8).

## Discussion

Dietary cholesterol overload is a major contributing factor for the development of cardiovascular and metabolic disorders. Hypercholesterolemia alters the physiological antioxidant abilities, resulting in ROS generation, and chronic inflammatory responses. Multiple lines of evidence support the notion that there is a linkage between cellular oxidative events and inflammation in various disorders induced by lipid discrepancies, particularly cardiovascular diseases 22. Under regular physiological status, the production of free radicals is limited and scavenged by the endogenous antioxidants. However, pathological conditions disrupt this balance in favor of ROS generation, resulting in oxidative stress. In the current study, the experimental observation documented that prolonged cholesterol overload triggers cardiac, hepatic and renal dysfunctions and over-production of ROS, which includes superoxide free radicals, hydrogen peroxide, and singlet oxygen. Markers of depleted antioxidant capacity such as low GSH levels as well as inhibited SOD, CAT and GPx activities were reported in the HCD group compared to normal animals. Free radical generation during HCD exposure was combined with cellular membranes lipid peroxidation with may harm functional cellular components. Our results come consistent with other studies that demonstrated augmented oxidative damage after HCD exposure 11, 23. The provoked lipid peroxidation indicates excessive ROS production that may exceed the detoxification capacity. Growing evidences suggest correlation between HCD and chronic inflammatory state. This assumption plays a crucial role in different diseases pathologies including diabetes and atherosclerosis. Studies have found that elevated cholesterol and fats values cannot initiate the pathological progression of pro-inflammatory cytokines 24. Moreover, the programmed cellular necrosis and its associated markers such as caspase 3 were found to be regulated by inflammatory mediators such as TNF-α 11. These cellular events alone with lipid peroxidation lead to defects in plasma membrane integrity, leakage of essential intracellular components, and damages of nucleic acids 25.

Presently, HCD group exhibited profound high levels of TNF-α, IL-1β, IL-6, NO caspase 3 and NF-κB alone with low IL-10 and PGE-2 levels, which indicates HCD-induced inflammatory response and DNA injury. Similar observation have been made by Zeng et al.^24^ who demonstrated that circulating free fatty acid caused cardiac damage *in vitro* and *in vivo* by activating NF-kB-mediated transcriptional signaling of antioxidant and inflammation genes, respectively.

Nowadays, phytochemical polyphenolic products are reported for use in multiple therapies. These natural products may protect against cardiovascular, ischemic, diabetes, hepatic and renal pathological conditions 26. BL is commonly promising polyphenolic compound with multiple therapeutic benefits. Several experimental studies reported the antioxidant and anti-inflammatory effects of BL in different biological systems. BL was found to protect against hypoxia re-oxygenation injury through recruitment of its oxidative and inflammatory cytokines suppressive effects 27. Another study reported that BL exhibited prominent ameliorative effects against oxidative and inflammatory injury of myocardial tissues in diabetic animals, which was mediated by PI3K/Akt signaling cascade 28. In addition, the hepatoprotective efficacy of BL was demonstrated in rodents with diabetic live injury 17.

Interestingly, Tsai et al found that BL attenuate the oxidized LDL-induced accumulation of cholesterol and foam cells formation in the subendothelial space, which suggest the potential role of BL against hypercholesterolemia 18. Our present findings are in agreement with these previous studies. BL corrected the elevated levels of TC, TG, and LDL-C, while enhanced HDL-C, which indicates the anti-hypercholesterolaemic effects. BL therapy showed cardio-protective effects confirmed by the alleviated CK-B, LDH, and CK-MB activities. Markers of liver toxicity including ALT and AST as well as nephrotoxicity markers such as creatinine and BUN were also restored by BL treatment. These cardiac, hepatic and renal protective effects were associated with repaired histological features in BL groups. Furthermore, BL treatment markedly re-activated the suppressed antioxidant enzymes SOD, CAT and GPx and suppressed the provoked lipid peroxidation in cardiac, hepatic and renal tissues. The unique chemical structure BL elucidates its pharmacological properties. Baicalein has tri-hydroxyl chemical groups at carbon number 5, 6 and 7. It also involves three saturated rings. These structural components are essential tool for free radical scavenger ability of most of flavones.

Limitations encountered in the current study include the unisexual testing of BL effects in hypercholesterolaemic male rats. This may interfere with assumption that gender metabolic and physiological differences may influence the protective effects of natural products against hypercholesterolemia and the associated molecular mechanisms. Moreover, the food consumption during the experimental period was not followed, which could have added to the explanation of the body weigh variations between different experimental groups.

## Conclusions

Our results showed that the supplementation of BL increases cardiovascular, renal and hepatic dysfunctions in experimentally induced hypercholesterolemia. The protective efficacy of BL was considerable in ameliorating cardiac, hepatic and renal oxidative injury *via* restoration of tissues regular histological features and antioxidant status. Regulation of pro-inflammatory and tissue apoptosis cellular mechanisms could contribute to BL protective mechanism against hypercholesterolemia and promotes its ability to attenuate ROS formation and antioxidant enzymes dysfunction.

## Figures and Tables

**Table 1:** Effect of BL on HCD-induced biochemical changes in serum measurements

Parameters	Control	HCD	BL(25)	BL(50)
TC (mg/dl)	47.95±7.42	112.94±28.51^***a^	92.87±11.77^*b^	76.64±5.57^**b^
TG (mg/dl)	21.03±9.24	59±12.65^***a^	44.55±12.33^*b^	37.61±5.96^**b^
HDL (mg/dl)	37.8±6.23	32.58±6.87	25.51±6.89	28.8±3.98
LDL (mg/dl)	10.38±3.05	47.16±10.59^***a^	42.44±4.37	36.56±5.72^*b^
LDH (U/L)	136.56±8.24	241.56±11.32^***a^	237.78±7.65	225.26±8.95^*b^
CK-B (U/L)	10.26±165	22.08±3.74^***a^	17.33±4.03^*b^	13.71±3.85^**b^
CK-MB (U/L)	20.54±3.31	44.19±11.48^***a^	28.68±8.06^**b^	23.44±7.70^***b^
Urea (mg/dl)	19.87±3.93	59.60±11.79^***a^	47.68±9.43^*b^	37.75±7.47^***b^
Creatinine (mg/dl)	2.06±0.67	6.18±2.00^***a^	4.94±1.60	3.91±1.26^*b^
AST (U/L)	36.67±6.94	54.17±4.67^***a^	45.56±8.34^*b^	38.89±5.24^**b^
ALT (U/L)	17.65±2.16	36.93±5.96^***a^	29.39±4.42^*b^	24.75±8.09^**b^

Total cholesterol (TC), triglycerides (TG), low density lipoprotein-cholesterol (LDL), high density lipoprotein-cholesterol (HD), creatine kinase-B (CK-B), lactate dehydrogenase (LDH), creatine kinase-MB (CK-MB), urea, creatinine, alanine aminotransferases (ALT) and aspartate aminotransferases (AST) levels. Statistically significant difference: ^a ***^p < 0.001 versus control group, ^b*^ p < 0.05, ^b**^ p < 0.01, ^b***^ p < 0.01 versus HCD group.

## Abbreviations

G): Baicalein (BL
